# Supplementary material for: The Parenting and Family Adjustment Scales (PAFAS) questionnaire: psychometric qualities of the parenting scale in two large Brazilian birth cohorts
Source: medRxiv. 2024 Sep 6:2024.09.06.24313039. Preprint. [Version 1] doi: 10.1101/2024.09.06.24313039 (PMC11398595; doi:10.1101/2024.09.06.24313039)
Supplement: Supplement 1 [file media-1.docx]

Supplementary table 1. Parenting and Family Adjustment Scales (PAFAS) original (18 items) and reduced (14 items) questionnaire in English and Portuguese

**Parenting and Family Adjustment Scale (PAFAS)**

***[Escala De Parentalidade e Ajustamento Familiar]***

Please read each statement and select a number 0, 1, 2 or 3 that indicates how true the statement was of you over the past four weeks. There are no right or wrong answers. Do not spend too much time on any statement.

*[Por favor, leia cada afirmação e assinale com um círculo o número da escala que mostra até que ponto cada afirmação é verdadeira, considerando as últimas quatro semanas. Não há respostas certas ou erradas. Não gaste muito tempo com cada afirmação.]*

|  | How true is this for you?  *[Até que ponto cada afirmação é verdadeira para você?]* | | | |
| --- | --- | --- | --- | --- |
|  | Not at all  *[Nem um pouco]* | A little  *[Um pouco (algum tempo)]* | Quite a lot *[Bastante (boa parte do tempo)]* | Very much *[Muito (a maior parte do tempo)]* |
| 1. If my child doesn’t do what they’re told to do, I give in and do it myself *[Se meu/minha filho/a não faz o que peço, eu desisto e eu mesma faço]* | 0 | 1 | 2 | 3 |
| 2. I give my child a treat, reward or fun activity for behaving well *[Eu dou uma guloseima, uma recompensa ou uma atividade divertida quando meu filho se comporta bem]* | 0 | 1 | 2 | 3 |
| 3. I follow through with a consequence (e.g. take away a toy) when my child misbehaves *[Quando meu filho se comporta mal, eu atribuo uma consequência planejada (por exemplo, retiro um brinquedo)]* | 0 | 1 | 2 | 3 |
| 4. I threaten something (e.g. to turn off TV) when my child misbehaves but I don’t follow through *[Quando meu/minha filho(a) se comporta mal eu ameaço (por exemplo desligar a televisão), mas não cumpro]* | 0 | 1 | 2 | 3 |
| 5. I shout or get angry with my child when they misbehave *[Eu grito ou fico braba com meu/minha filho(a) quando ele(a) se comporta muito mal]* | 0 | 1 | 2 | 3 |
| 6. I praise my child when they behave well *[Eu elogio meu/minha filho(a) quando ele/ela se comporta bem]* | 0 | 1 | 2 | 3 |
| 7. I try to make my child feel bad (e.g. guilt or shame) for misbehaving to teach them a lesson *[Eu tento fazer meu/minha filho(a) se sentir mal (por exemplo culpado/a ou envergonhado/a) por se comportar mal, para lhe ensinar uma lição]* | 0 | 1 | 2 | 3 |
| 8. I give my child attention (e.g. a hug, wink, smile or kiss) when they behave well *[Eu dou atenção a meu/minha filho(a) como um abraço, uma piscada de olho, um sorriso, ou um beijo quando ele/ela se comporta bem]* | 0 | 1 | 2 | 3 |
| 9. I spank (smack) my child when they misbehave *[Eu dou um palmada no/a meu/minha filho(a) quando ele(a) se comporta mal]* | 0 | 1 | 2 | 3 |
| 10. I argue with my child about their behaviour/attitude *[Eu discuto com meu filho sobre seu comportamento/attitude]* | 0 | 1 | 2 | 3 |
| 11. I deal with my child’s misbehaviour the same way all the time *[Eu lido com o mau comportamento do meu filho da mesma maneira, o tempo todo]* | 0 | 1 | 2 | 3 |
| 12. I give my child what they want when they get angry or upset *[Eu dou a meu/minha filho(a) o que ele/ela quer quando ele(a) fica com raiva ou chateado/a]* | 0 | 1 | 2 | 3 |
| 13. I get annoyed with my child *[Eu fico irritada com o(a) meu/minha filho(a)]* | 0 | 1 | 2 | 3 |
| 14. I chat/talk with my child *[Eu converso com meu/minha filho(a)]* | 0 | 1 | 2 | 3 |
| 15. I enjoy giving my child hugs, kisses and cuddles *[Eu gosto de dar abraços, beijos e fazer carinho no(a) meu/minha filho(a)]* | 0 | 1 | 2 | 3 |
| 16. I am proud of my child *[Eu sou orgulhosa do/da meu/minha filho(a)]* | 0 | 1 | 2 | 3 |
| 17. I enjoy spending time with my child *[Eu gosto de passar o tempo com o/a meu/minha filho(a)]* | 0 | 1 | 2 | 3 |
| 18. I have a good relationship with my child *[Eu tenho um bom relacionamento com o/a meu/minha filho(a)]* | 0 | 1 | 2 | 3 |

Note: Translation of the instrument conducted by Correia et al., 2024, authorized by the authors of PAFAS. Grey cells were removed in the reduced version.
